# Mutations in *TP53* increase the risk of *SOX2* copy number alterations and silencing of *TP53* reduces SOX2 expression in non-small cell lung cancer

**DOI:** 10.1186/s12885-016-2061-3

**Published:** 2016-01-19

**Authors:** Johanna Samulin Erdem, Vidar Skaug, Per Bakke, Amund Gulsvik, Aage Haugen, Shanbeh Zienolddiny

**Affiliations:** Department of Biological and Chemical Work Environment, National Institute of Occupational Health, PO Box 8149 Dep, , N-0033 Oslo, Norway; Department of Clinical Science, University of Bergen, Bergen, Norway

**Keywords:** SRY (sex determining region Y)-box 2, NSCLC, *TP53* mutation, Copy number alteration, Hsa-miR-14

## Abstract

**Background:**

Amplifications of the transcription factor, SRY (sex determining region Y)-box 2 (*SOX2*), are common in non-small cell lung cancer (NSCLC). SOX2 signaling is important in maintaining the stem cell-like phenotype of cancer cells and contributes to the pathogenesis of lung cancer. TP53 is known to inhibit gene amplifications and to repress many stem cell-associated genes following DNA damage. The aim of this study was to investigate if *TP53* mutational status affected *SOX2* copy number variation and gene expression in early-stage NSCLC patients; moreover, to assess if TP53 regulates SOX2 expression in human lung cancer cells.

**Methods:**

258 early-stage lung cancer patients were included in the study. Exons 4–9 in the *TP53* gene were sequenced for mutations in tumor tissues. *SOX2* copy number as well as TP53 and SOX2 gene expression were analyzed in tumor and in adjacent non-tumorous tissues by qPCR. *TP53* and *SOX2* were silenced using gene-specific siRNAs in human lung adenocarcinoma A427 cells, and the expression of TP53, SOX2 and subset of selected miRNAs was analyzed by qPCR. The odds ratios (ORs) for associations between copy number variation and lung cancer were estimated by conditional logistic regression, and the correlation between gene status and clinicopathological characteristics was assessed by Chi-square or Fisher’s exact test. Gene expression data was analyzed using nonparametric Mann–Whitney test.

**Results:**

*TP53* mutations were associated with an increased risk of acquiring a *SOX2* copy number alteration (OR = 2.08, 95 % CI: 1.14–3.79, *p* = 0.017), which was more frequently occurring in tumor tissues (34 %) than in adjacent non-tumorous tissues (3 %). Moreover, SOX2 and TP53 expression levels were strongly correlated in tumor tissues. *In vitro* studies showed that a reduction in TP53 was associated with decreased SOX2 expression in A427 cells. Furthermore, *TP53* knockdown reduced the miRNA hsa-miR-145, which has previously been shown to regulate SOX2 expression.

**Conclusions:**

TP53 signaling may be important in the regulation of *SOX2* copy number and expression in NSCLC tumors, and the miRNA hsa-miR-145-5p may be one potential driver. This prompts for further studies on the mechanisms behind the TP53-induced regulation of SOX2 expression and the possible importance of hsa-miR-145 in lung cancer.

**Electronic supplementary material:**

The online version of this article (doi:10.1186/s12885-016-2061-3) contains supplementary material, which is available to authorized users.

## Background

Lung cancer is the most frequent cause of cancer-related mortality worldwide, leading to an estimated 1.4 million deaths in 2010 [1]. Smoking, occupational and environmental exposures to chemicals are major causes of lung cancer. Lung cancer constitutes a heterogeneous disease in regard to clinical presentation, pathological features and biological behavior. The majority of cases are non-small cell lung carcinomas (NSCLC), which comprises adenocarcinoma, squamous cell carcinoma and large cell carcinoma. The genomic alterations occurring in lung carcinomas are very complex [2–5]. However, alterations in the *TP53* gene are among the most significant genetic events in lung cancers [6], often occurring as a response to DNA damage caused by exposure to a variety of genotoxic agents such as polycyclic aromatic hydrocarbons (PAHs) [7]. Mutations in the *TP53* gene increase the risk for chromosomal rearrangements, such as copy number alterations, which are involved in the development and progression of many human malignancies including lung cancer [8]. Amplifications or deletions in the fragile sites harboring important transcription factors may further advance the process of carcinogenesis [9].

The transcription factor SRY (sex determining region Y)-box 2, encoded by the *SOX2* gene located at the 3q25-27 region, is often altered in NSCLC [10, 11]. SOX2 has a crucial role in maintaining the stem cell-like phenotype in cancer cells [12, 13] and contributes to the pathogenesis of lung cancer by controlling cell proliferation and malignant transformation [11]. In lung cancer, *SOX2* gene amplification and consequent increased expression occur most frequently in squamous cell carcinoma [14, 15] and to a lesser extent in adenocarcinoma [14, 16]. Interestingly, TP53 has been reported to regulate SOX2 expression in embryonic stem cells [17] and recently also in the H1299 lung carcinoma cell line [18]. miRNAs are important mediators of TP53-signaling and in embryonic stem cells TP53 represses SOX2 expression through the activation of hsa-miR-145 [19–21]. This is of interest as a recent study showed that low levels of hsa-miR-145 are associated with unfavorable prognosis in NSCLC [22].

Given the crucial role of TP53 and SOX2 in lung cancer and their known association in stem cell development, we hypothesize that TP53 may have a regulatory effect on SOX2 in lung cancer. Thus, effects of *TP53* mutations on *SOX2* copy number alterations were studied in lung cancer tumors and correlation between the gene expression levels investigated. Furthermore, effects of *TP53* silencing on SOX2 mRNA levels were evaluated and the possibility of miRNAs as downstream regulators was assessed.

## Methods

### Cases

Early-stage lung cancer patients (*n* = 258) were Caucasians of Norwegian origin admitted to Haukeland University Hospital in Bergen between 1988 and 1994, for primary surgery. The patients were enrolled in the study, whenever practically feasible, and informed written consent covering analysis of molecular and genetic markers was signed by the patients prior to surgery. The subjects included in this manuscript are a subgroup recruited into the project “lung cancer genetics” at our institute. The project has been approved by the Regional Committee for Medical and Health Research Ethics in South Norway in accordance with the WMA Declaration of Helsinki. The ethical approval covered access to the NSCLC databank. The characteristics of the patients included in the study are summarized in Table 1. Samples of adjacent non-tumorous lung tissue, confirmed by histology, were cut from the lobectomi specimens at the time of surgery. Tumor histology was confirmed by an experienced pathologist and samples containing ≥80 % of tumor cells were analyzed in the study. After resection tumor and non-tumorous tissues were snap-frozen in liquid nitrogen and kept at −80 °C until further processing.

**Table 1:** Characteristics of patients (*n* = 258)

*Age*	
(Mean ± SD)	63.9 ± 10.1
(Median, min-max)	66 (25–82)
* Gender* (male: female)	183/74
* Smokers/Non-smokers*	222/22
*No cigarettes per day*	
(Mean ± SD)	15.3 ± 7.9
(Median, min-max)	14 (2–50)
*Total years of smoking*	
(Mean ± SD)	41.3 ± 11.6
(Median, min-max)	43 (2–69)
Total pack-years*	
(Mean ± SD)	31.6 ± 18.2
(Median, min-max)	28 (1–113)
*Histology*	
Adenocarcinoma	118 (45.7 %)
Squamous cell carcinoma	103 (39.9 %)
Large cell carcinoma	36 (13.9 %)
Others	1
* PAH-DNA adducts* ^*†*^	
(Mean ± SD)	12.3 ± 8.9
(Median, min-max)	10 (1–46)
* TP53 mutated/WT*	134/101

^*^Pack-years indicates the number of cigarettes smoked per day x number of years smoked/20. ^*†*^PAH-DNA adducts per 10^8^ nucleotides

### Copy number analyses by quantitative PCR

DNA was extracted from frozen lung tissue samples using standard proteinase K digestion followed by phenol–chloroform extraction and ethanol precipitation. Control DNA was isolated from blood samples by Flexigene DNA kit (Qiagen, Hilden, Germany). *SOX2* copy number alterations were evaluated by quantitative real-time PCR (qPCR) using SYBR Green I technology on an ABI PRISM® 7900HT Fast PCR System (Applied Biosystems, Thermo Scientific, Waltham, MA, USA), as described elsewhere [23, 24]. The multicopy gene *FTH1* was used as reference gene. Primer sequences were: *SOX2* forward primer, 5’-GCTCTTGGCTCCATGGGTTC-3’, reverse primer, 5’-GCTGATCATGTCCCGGAGGT-3’, *FTH1* forward primer, 5’-GATGATGTGGCTTTGAAGAACTTTGCCA-3’, reverse primer, 5’-CACCTCGTTGGTTCTGCAGCTTCATCA-3’. Primer specificity was determined by melting point analysis. qPCR was performed using 20 ng template DNA in a total volume of 10 μL containing PerfeCTa SYBR Green FastMix, ROX (QuantaBioSciences, Gaithersburg, MD, USA). Cycling conditions were: 95 °C, 2 min followed by 40 cycles of 95 °C, 10 sec and 60 °C, 45 sec. The PCR was run in duplicates using a relative standard curve approach. The standard curve was generated by performing serial dilutions of plasmid DNA containing one copy of the area of interest for each of the assayed genes. pUC57 plasmid DNA (GenScript, Piscataway, NJ,USA) was added to each standard to maintain a constant amount of total DNA per reaction tube. Only R^2^ values above 0.99 were accepted and data was quality controlled in accordance with previous recommendations [23]. For confirmation, analysis of >5 % of all samples was repeated. Control DNA isolated from cancer-free individuals was used to normalize for plate variability. Copy numbers below 1.5 and above 2.5 were defined as deleted and amplified, respectively.

### Analysis of frequency of TP53 mutations and PAH-DNA adduct levels

To assess the frequency of *TP53* mutations in tumor tissues DNA was screened either by single-strand conformational polymorphism or denaturating capillary electrophoresis as previously described, covering exons 4 to 9 of the *TP53* gene, and DNA samples with alterations were sequenced [25, 26]. PAH-DNA adduct levels were determined in non-tumorous lung tissue by ^32^P-postlabelling as previously published [27]. The patients were divided into two groups on the basis of having levels greater than or less than the median (9.91) of DNA adducts per 10^8^ nucleotides.

### Cell culture and RNA knockdown

Lung adenocarcinoma A427 cells were maintained in RPMI-1640 medium (Thermo Scientific) with 10 % FCS (Thermo Scientific), and penicillin/streptomycin (Biowest SAS, Nuaillé, France) in 5 % CO_2_ at 37 °C. The cells were passaged every third day and passage numbers between 25 and 30 were used in all experiments. In RNA knockdown experiments 100000 cells/mL were seeded in 2 mL growth medium without penicillin/streptomycin in 6-well plates. For protein isolation 200000 cells/mL were seeded in 10 mL medium without penicillin/streptomycin in 10 cm^2^ plates. siRNA targeting human *TP53*, *SOX2* and non-target (control) were purchased from Applied Biosystems (Thermo Scientific). Transfections were performed 24 h after seeding using 10 nM siRNA and Lipofectamin RNAiMAX reagent (Invitrogen, Thermo Scientific) according to manufacturer’s instructions. The cells were harvested 48 h after transfection to analyze RNA, miRNA and protein expression.

### Western blotting

Nuclear proteins were extracted from knockdown and control A427 cells using NE-PER Nuclear and Cytoplasmic extraction reagents (Thermo Scientific, Rockford, IL, USA). Protein concentrations were determined by BCA Protein Assay (Thermo Scientific) and 40 µg lysate was separated by 10 % SDS-PAGE and transfer to a Immobilon PVDF membrane (Millipore, Bedford, MA, USA). To prevent non-specific background binding, the membranes were incubated with 5 % non-fat milk in Tris-buffered saline with 0.05 % Tween-20 (TBST) for 1 h at room temperature. Membranes were incubated with primary antibodies against SOX2 and TP53 (Cell Signaling technology Inc., Beverly, MA, USA) over night at 4 °C and against GAPDH (Santa Cruz Biotechnology, Santa Cruz, CA, USA) for 1 h at room temperature. After washing three times with TBST, the membranes were incubated with secondary anti-rabbit antibody (Cell Signaling) for 1 h at room temperature and proteins visualized by SuperSignal West Pico Chemiluminescent Substrate (Thermo Scientific). Semiquantitative densitometry analysis were performed using Image J.

### Gene expression analysis

Total RNA was isolated from frozen lung tissue samples using standard Trizol extraction and total RNA from transfected cells was isolated using NucleoSpin miRNA (Macherey-Nagel, Duren, Germany). RNA was DNase treated with DNA-free kit (Ambion, Thermo Scientific) and its quality was assessed by 2100 Bioanalyzer (Agilent Technologies, Santa Clara, CA, USA). For gene expression analysis total RNA was reversed transcribed using qScript cDNA Supermix or qScript cDNA synthesis kit (Quanta BioSciences) for tissue samples and cells respectively. Beta-Actin (*ACTB*) was used as reference gene. Primer sequences were: SOX2 forward primer, 5’-GGGGAAAGTAGTTTGCTGCC-3’, reverse primer, 5’-CGCCGCCGATGATTGTTATT-3’, TP53 forward primer, 5’-CCATCCTCACCATCATCACA-3’, reverse primer, 5’-CACAAACACGCACCTCAAAG-3’, ACTB forward primer, 5’-GCGAGAAGATGACCCAGATCA-3’, reverse primer, 5’-GATAGCACAGCCTGGATAGCAA-3’. TargetScan 6.2 (http://www.targetscan.org/) was used to predict conserved miRNA binding sites in the *SOX2* gene. Of these five previously known targets of TP53; hsa-miR-145-5p, hsa-miR-145-3p, hsa-miR-200b-3p, hsa-miR-200c-3p, hsa-miR-429-1p; were selected for further analysis. Accordingly, total RNA was reversed transcribed using qScript microRNA cDNA Synthesis Kit (Quanta BioSciences) and expression was analyzed using commercial primers (Integrated DNA Technologies, Leuven, Belgium). SNO and RNU6 were used as references. Expression was evaluated by qPCR using SYBR Green I technology on an ABI PRISM® 7900HT Fast Real-Time PCR System (Applied Biosystems, Thermo Scientific). Primer specificity was determined by melting point analysis.

### Statistical analysis

Statistical analyses were carried out using IBM SPSS software version 22.0. Associations between copy number variation and lung cancer were estimated by odds ratios (ORs) and their 95 % confidence intervals (CIs) from conditional logistic regression adjusted for age, gender, total pack-years and tumor histology. The total number of patients included in the study was 258. However, for some patients, data on smoking status, gender or age was missing, and for five patients CNV data was not successfully obtained, thus giving a reduced number of patients included in the final statistical analysis. Effects of clinicopathological data on gene status were assessed by Chi-square or Fisher’s exact test for categorical variables and by nonparametric tests for ordinal variables. Cancer free survival was obtained from 241 patients by Kaplan Meier analysis. Expression data was analyzed using nonparametric Mann–Whitney test or one-way ANOVA. *p* < 0.05 was considered significant.

## Results

### SOX2 copy number alterations are frequent in NSCLC tumors

**Fig. 1:**
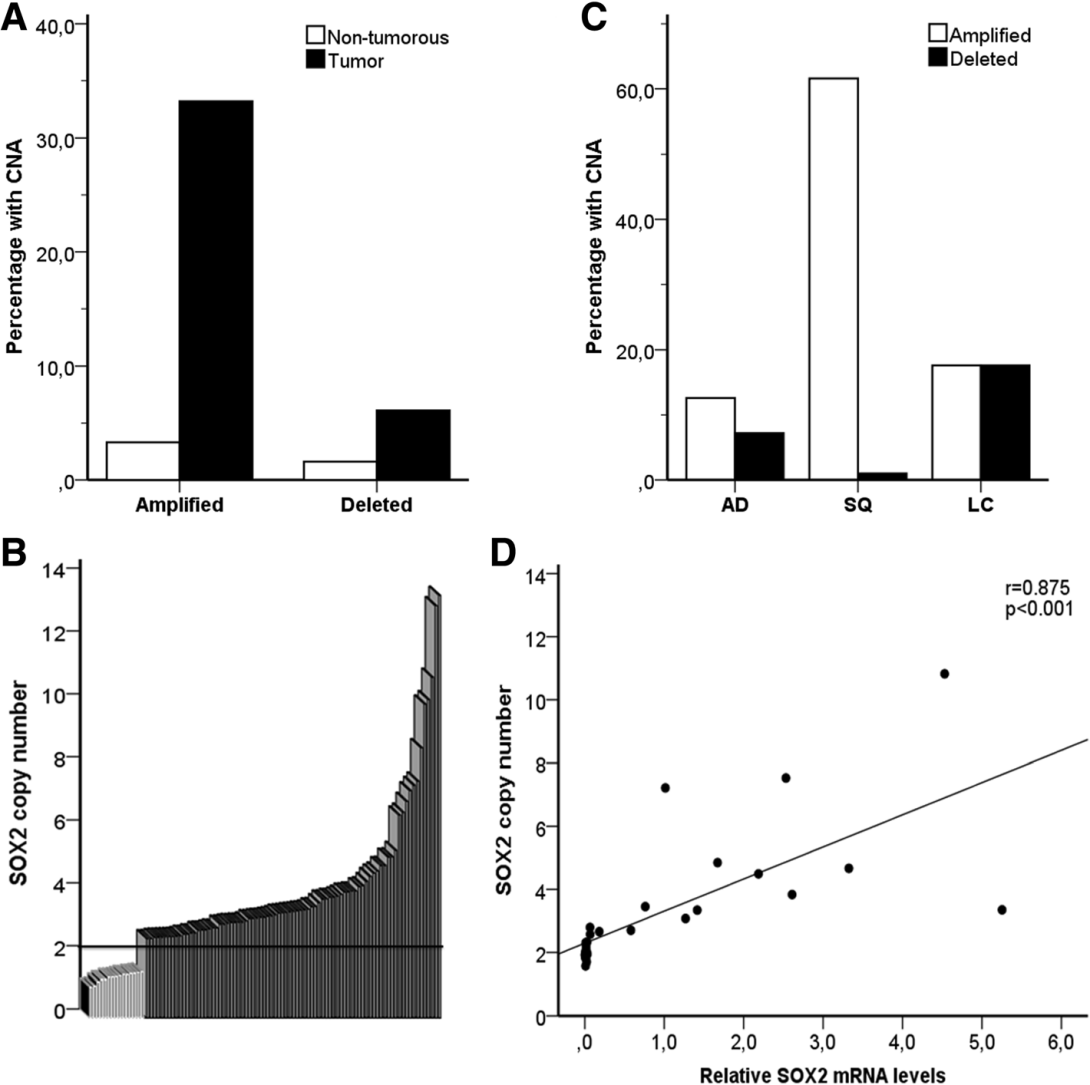
*SOX2* copy number alterations in lung tumors (*n* = 244). **a** Percentage of *SOX2* gene amplifications and deletions in lung tumors and non-tumorous tissues. **b** Distribution of *SOX2* copy numbers in lung tumors. More than 2.5 copy numbers were defined as amplifications, whereas less than 1.5 copy numbers were defined as deletions. **c** Distribution of copy number alterations between NSCLC of different histology. AD: adenocarcinoma, SQ: squamous cell carcinoma and LC: large cell carcinoma. **d** Correlation between the relative genomic *SOX2* levels and its mRNA expression. *r* = 0.875, *p* < 0.001 was obtained from Spearman's Rank-Order Correlation (*n* = 30)

Analyses of gene copy numbers were performed on DNA from paired non-tumorous and tumor tissue of the same patient in order to evaluate whether genomic variations in *SOX2* may be associated with lung tumor development. The *SOX2* copy number alterations were more frequent in tumors than in non-tumorous tissues of NSCLC patients (Fig. 1a). The odds for acquiring a copy alteration in *SOX2* were 12.6-fold higher (95 % CI: 5.8–27.1) in tumors than in non-tumorous tissues. *SOX2* gene amplifications were observed in 33.6 % of tumors compared with 3.2 % in the adjacent non-tumorous tissues (OR: 15.7, 95 % CI: 6.3–39.1, *p* < 0.001). A low occurrence of *SOX2* gene deletions was also observed in 6.1 % of tumors (OR: 6.0, 95 % CI: 1.6–23.0, *p* = 0.009). Amplifications of >5 copies were observed in 15 of 81 tumors with a *SOX2* amplification (Fig. 1b), and *SOX2* copy number alterations were most frequent in squamous cell carcinoma (Fig. 1c). Moreover, changes in *SOX2* gene status were more frequent in smokers than non-smokers (*p* = 0.015, Additional file 1: Table S1). SOX2 mRNA expression levels were investigated in 15 tumor and 15 non-tumorous tissue samples, and a strong correlation, (*r* = 0.875, *p* = 0.001), was observed between the *SOX2* copy numbers and the relative SOX2 mRNA expression level (Fig. 1d). *SOX2* copy number alterations did not affect the survival of the patients (data not shown).

### Mutations in TP53 gene increase frequency of SOX2 copy number alterations

**Fig. 2:**
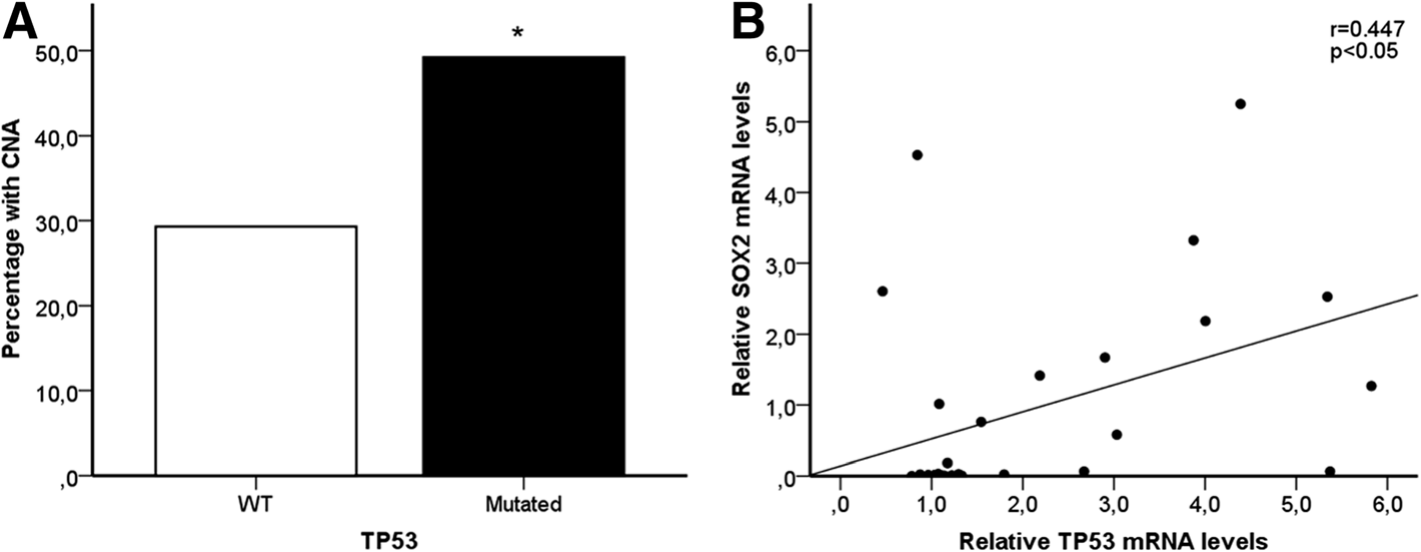
**a** *TP53* gene status in tumor tissue of patients and its association with copy number alterations (CNA) in the *SOX2* gene. **p = 0.017, was obtained from unconditional logistic regression, adjusted for age, gender, total pack-years and histology (*n* = 229). **b** Correlation between the relative SOX2 and TP53 mRNA expression. *r* = 0.447, *p* = 0.013 was obtained from Spearman's Rank-Order Correlation (*n* = 30)

The effects of *TP53* mutations on *SOX2* copy number were investigated in 229 tumor tissues of the NSCLC patients. Of these, 56.8 % had at least one mutation in their lung tumors. Patients with mutated *TP53* had a significantly higher odds of acquiring a copy number variation in *SOX2* (OR = 2.08, 95 % CI: 1.14-3.79, *p* = 0.017) than patients without *TP53* mutations (Fig. 2a). Furthermore, a positive correlation (*r* = 0.447, *p* = 0.013) was observed between TP53 and SOX2 mRNA expression levels in a subset of cancer patients (*n* = 30, Fig. 2b). *TP53* mutations are often occurring as a consequence of DNA-adduct formation following exposure to genotoxic carcinogens such as PAHs in cigarette smoke. However, increased PAH-adduct levels did not increase the occurrence of *SOX2* copy number alterations in our material (data not shown), despite a higher frequency of *SOX2* gene alterations in smokers compared to the non-smoking NSCLC patients.

### TP53 affects SOX2 expression and hsa-miRNA-145-5p levels in human lung cells in vitro

**Fig. 3:**
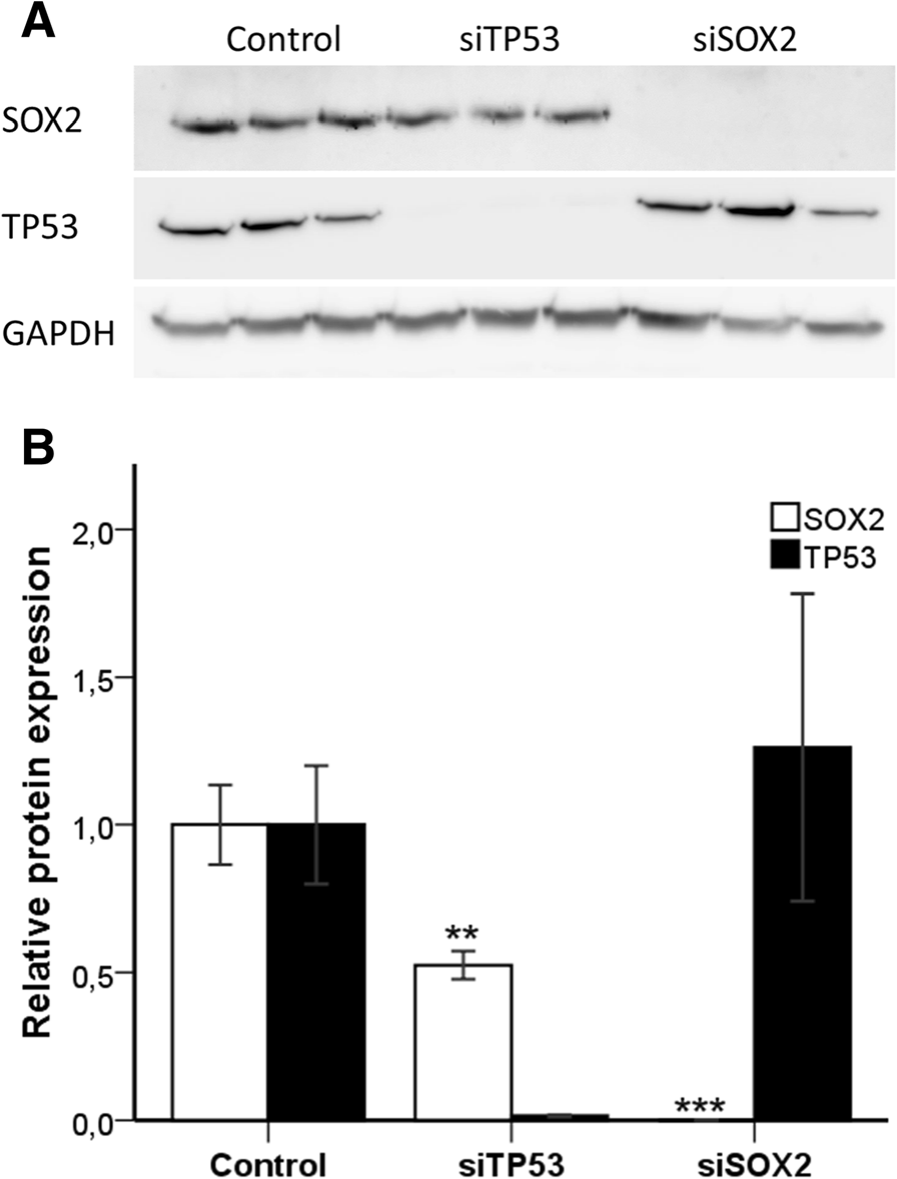
**a** Efficient SOX2 and TP53 knockdown was confirmed by western blotting. *SOX2* and *TP53* were silenced using siRNA technology. After 48 h nuclear lysates were isolated from knockdown and control cells. 40 µg lysate/lane was resolved in a SDS-PAGE and membrane analysed for expression of SOX2, TP53 and GAPDH (loading control) by western blotting. **b** Protein expression was semiquantitatively analysed by densitometry using Image J. Bars indicate mean ± SE, *n* = 3. ***indicating *p* < 0.001 and **indicating *p* < 0.01 was obtained from one-way ANOVA

**Fig. 4:**
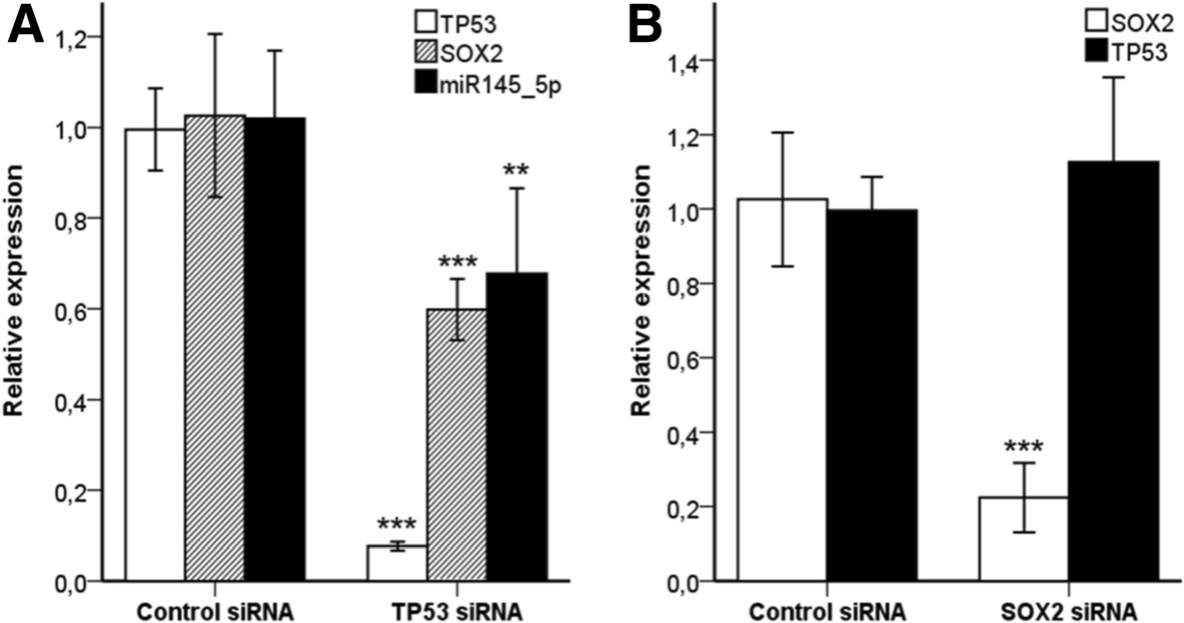
Effects of silencing of the *TP53* and *SOX2* genes on the expression of SOX2, TP53 and hsa-miRNA-145-5p in pulmonary adenocarcinoma A427 cells. **a** *TP53* was silenced using siRNA technology and expression of SOX2 and hsa-miR-145-5p was analyzed by qPCR. Bars indicate mean ± SE, n = 9 and n = 12 for SOX2 and hsa-miR-145-5p, respectively. **b** *SOX2* was silenced using siRNA technology and expression of TP53 was analyzed by qPCR. Bars indicate mean ± SE, *n* = 9. ***indicating *p* < 0.001 and **indicating *p* < 0.01 was obtained from nonparametric Mann–Whitney test

To investigate the effect of TP53 on SOX2 expression in NSCLC, the *TP53* gene was silenced in the lung adenocarcinoma A427 cell line using siRNA. Effective knockdown of TP53 and SOX2 proteins after 48 h was confirmed in three independent experiments by western blotting (Fig. 3a). In *TP53* knockdown cells a significant 40 % reduction in SOX2 mRNA and protein expression was observed (*p* = 0.001, Fig. 4a and *p* = 0.01, Fig. 3b). *SOX2* knockdown did not affect the expression of TP53 (Fig. 4b). Moreover, hsa-miR-145-5p expression was significantly reduced in *TP53* knockdown cells (*p* = 0.004, Fig. 4a). In contrast, the levels of hsa-miR-145-3p, hsa-miR-200b and hsa-miR-200c remained unchanged in *TP53* knockdown cells, whereas hsa-miR-429 was not expressed in these cells (data not shown).

## Discussion

SOX2 has a crucial role in maintaining the stem cell-like phenotype in cancer cells [12, 13] and its overexpression is generally associated with aggressive disease and poor outcome in several different tumor types [28–32]. *SOX2* has been identified as the driving gene of the 3q25-27 amplicon, which is common in most NSCLC cases and contributes to the pathogenesis of lung cancer by controlling cell proliferation and malignant transformation [11]. Comparison of tumor tissue with non-tumorous tissue obtained from the same patients confirmed that tumor tissues had significantly higher odds of acquiring a copy number alteration in the *SOX2* gene. Surprisingly, *SOX2* copy number deletions were detected in a subset of tumor samples in this cohort. Deletions in *SOX2* copy number have not previously been reported in NSCLC and the importance of this finding remains to be elucidated. *SOX2* gene amplifications were observed in 34 % of tumors in this study. SOX2 has been shown to be highly expressed in approximately 90 % of pulmonary squamous cell carcinoma and to a lesser extent in adenocarcinoma [16] and several studies have previously identified *SOX2* gene amplifications in lung squamous cell carcinomas with reported frequencies from 20 % to 60 % [13, 15, 33–36]. Here, we observed higher frequency of *SOX2* copy number amplifications in squamous cell carcinoma tumors (59 %) than in adeno- (20 %) or large cell carcinoma (34 %). These data are in line with previous reports indicating a role for SOX2 in carcinogenesis. Accordingly, SOX2 affects tumorigenesis and its overexpression increases cell migration and colony formation, while its knockdown impairs cell growth and suppresses metastasis of lung cancer cells [35, 37–39]. Increased SOX2 expression is associated with metastatic progression [40] and *SOX2* gene amplifications are more frequent in high-grade than low-grade lesions [41]. Moreover, conditional homozygous overexpression of SOX2 in murine Clara cells induces bronchial epithelial hyperplasia with progression to lung cancer in approximately 50 % of the animals [42]. Taken together, these results support the hypothesis that SOX2 may act as an oncogene in lung carcinogenesis.

The prognostic role of SOX2 in NSCLC remains uncertain as conflicting data has been reported. *SOX2* amplifications and overexpression have been reported as predictors of prolonged survival in squamous cell carcinomas [15, 43]. On contrary, SOX2 expression is suggested a poor prognosis predictor in stage I lung adenocarcinoma [44]. Wilbertz et al. investigated *SOX2* amplifications and expression in two cohorts and reported no association between SOX2 expression and survival in lung adenocarcinoma; however, observed a decreased survival in patients with low-level amplifications in one of the cohorts [15]. Differences in biological properties of tumor-initiating cancer stem cells in squamous cell carcinoma compared to lung or breast adenocarcinoma have been suggested as an explanation to the different observed associations between *SOX2* gene amplifications and prognosis [45]. Recently, several studies have showed improved outcome as a consequence of *SOX2* gene amplification in NSCLC, independent of histological subtype [14, 46, 47]. In our study, no significant effect on survival rates could be demonstrated, but this may be due to limited number of patients investigated. Moreover, differences in patient clinical characteristcs, methodology and scoring criteria might explain some of the reported discrepancies. Clearly, more studies are needed to elucidate the role of SOX2 in lung cancer progression and prognosis.

TP53 has been demonstrated to down-regulate several stem cell-associated genes, including *SOX2*, following DNA damage in embryonic stem cells [17]. Moreover, a recent report indicated that induction of TP53 led to repressed SOX2 expression in NSCLC cells [18]. Since genetic alterations in the *TP53* gene are significant events in lung cancers [6], and mutations in the *TP53* gene increase the risk for chromosomal rearrangements such as copy number alterations [8], these early reports open up the possibility of TP53 as an important regulator of SOX2 in NSCLC. To address this, the effects of *TP53* mutational status on *SOX2* copy number and expression levels were studied in NSCLC patients. Interestingly, patients with at least one mutation in their lung tumors possessed a 2-fold higher risk of having a copy number variation in *SOX2*. This is to our knowledge the first study to report an association between *TP53* mutations and *SOX2* copy number alterations. However, individuals carrying *TP53* mutations show generally higher levels of germline copy number variation [48]. Furthermore, smoking is known to increase the risk of acquiring *TP53* mutations [49–51] and, similarly, we and others [34, 36] have demonstrated that smokers have a higher risk of acquiring *SOX2* copy number alterations in tumors. Hence, *TP53* mutations may contribute to *SOX2* copy number alterations in lung cancer patients. Moreover, a correlation between TP53 and SOX2 expression levels was observed in NSCLC tumors. To further investigate this relationship, silencing of *TP53* and *SOX2* was performed in the lung adenocarcinoma cell line A427. *TP53* knockdown reduced SOX2 mRNA and protein expression, whereas *SOX2* knockdown did not affect TP53 expression levels, indicating a regulatory role of TP53 on SOX2 in NSCLC. Considering the role of SOX2 in tumorigenesis it could be expected that *TP53* knockdown would increase SOX2 expression. Indeed, Chen et al. have previously shown a reduction in SOX2 expression following TP53 induction in the *TP53*-null NSCLC cell line H1299 [18]. These discrepancies as well as reports on conflicting effects on NSCLC prognosis following *SOX2* copy number amplification indicate that the role of SOX2 in NSCLC is far more complex than can be explained by histology or *TP53* status only.

There is a need to further investigate the mechanisms of TP53 regulation of SOX2. To address this, the possible involvement of miRNAs was investigated. This is of interest as a more prominent role of miRNAs in cancer development has emerged during recent years and TP53 regulates the expression of several genes through miRNAs [52, 53]. Hsa-miR-145 is an interesting candidate as TP53 activates expression of hsa-miR-145 which in turn represses SOX2 expression in embryonic stem cells [19–21]. Hsa-miR-145 expression has also been suggested as a novel marker responsible for relapse in surgically treated NSCLC [22]. Campayo et al. demonstrated that TP53 may play a role in modulating hsa-miR-145 expression in NSCLC, and that patients with mutations in *TP53* and low hsa-miR-145 levels had lower survival in respect to patients with *TP53* wild-type or only mutations in *TP53* [22]. We here demonstrated that *TP53* knockdown repressed hsa-miR-145-5p expression in the lung adenocarcinoma cell line A427, whereas the other miRNAs investigated were unaffected. miRNAs have previously been believed to affect gene expression modulation only by negative regulation of target mRNA. Increasing evidence now indicate that miRNAs oscillate between repression and induction of gene expression in response to specific cellular conditions and cofactors [54]. Based on our findings, we suggest a possible role of hsa-miR-145 as an inducer of SOX2 expression in NSCLC. It is known that hsa-miR-145 exhibits opposite effects on RNA regulation in different cell types. Indeed, hsa-miR-145 mediates gene upregulation during muscle differentiation [55] and gene downregulation in osteosarcoma [56]. Other approaches to further investigate the possible regulation of TP53 on SOX2 in several different lung cancer cell lines and in different cellular states are clearly needed, as well as additional studies to fully understand the complexity of hsa-miR-145 signaling in lung cancer. Moreover, hsa-miR-638 has recently come into focus as another promising candidate, as it has been shown that hsa-miR-638 regulates SOX2 in NSCLC [57]. Interestingly, hsa-miR-638 is known as a TP53-targeting miRNA [58], further illustrating the complexity of this signaling.

## Conclusions

*TP53* mutations were associated with an increased risk of acquiring a *SOX2* copy number amplification in NSCLC. Furthermore, SOX2 and TP53 expression were correlated in lung tumors and reduction in TP53 resulted in decreased SOX2 and hsa-miR-145 expression in lung adenocarcinoma cells. As hsa-miR-145 has previously been shown to regulate SOX2 expression, we propose that one mechanism for the TP53 regulation of SOX2 may be through hsa-miR-145. This prompts for further studies on the mechanisms behind TP53-induced regulation of SOX2 expression and the possible importance of hsa-miR-145 in lung cancer development.

## Additional file

Additional file 1: Table S1. Association between clinicopathological data and *SOX2* gene status in NSCLC tumors. (PDF 101 kb)

## Abbreviations

NSCLC: Non-small cell lung cancer
OR: Odds ratio
PAH: Polycyclic aromatic hydrocarbons

## References

[1] Jemal A, Bray F, Center MM, Ferlay J, Ward E, Forman D (2011). Global cancer statistics. CA Cancer J Clin..

[2] Staaf J, Isaksson S, Karlsson A, Jonsson M, Johansson L, Jonsson P (2013). Landscape of somatic allelic imbalances and copy number alterations in human lung carcinoma. Int J Cancer.

[3] Seo JS, Ju YS, Lee WC, Shin JY, Lee JK, Bleazard T (2012). The transcriptional landscape and mutational profile of lung adenocarcinoma. Genome Res..

[4] Iwakawa R, Takenaka M, Kohno T, Shimada Y, Totoki Y, Shibata T, Tsuta K, Nishikawa R, Noguchi M, Sato-Otsubo A *et al*. Genome-wide identification of genes with amplification and/or fusion in small cell lung cancer. Genes Chromosomes Cancer. 2013;52:802-16. 10.1002/gcc.22076 PMC3806277 23716474

[5] Cancer Genome Atlas Research Network (2012). Comprehensive genomic characterization of squamous cell lung cancers. Nature.

[6] Whibley C, Pharoah PD, Hollstein M (2009). p53 polymorphisms: cancer implications. Nat Rev Cancer..

[7] Henkler F, Stolpmann K, Luch A (2012). Exposure to polycyclic aromatic hydrocarbons: bulky DNA adducts and cellular responses. EXS..

[8] Llanos S, Efeyan A, Monsech J, Dominguez O, Serrano M (2006). A High-Throughput Loss-of-Function Screening Identifies Novel p53 Regulators. Cell Cycle..

[9] Dillon LW, Burrow AA, Wang Y-H (2010). DNA Instability at Chromosomal Fragile Sites in Cancer. Current Genomics..

[10] Dehan E, Ben-Dor A, Liao W, Lipson D, Frimer H, Rienstein S (2007). Chromosomal aberrations and gene expression profiles in non-small cell lung cancer. Lung Cancer..

[11] Balsara BR, Testa JR (2002). Chromosomal imbalances in human lung cancer. Oncogene..

[12] Gontan C, de Munck A, Vermeij M, Grosveld F, Tibboel D, Rottier R (2008). Sox2 is important for two crucial processes in lung development: branching morphogenesis and epithelial cell differentiation. Dev Biol..

[13] Yuan P, Kadara H, Behrens C, Tang X, Woods D, Solis LM (2010). Sex determining region Y-Box 2 (SOX2) is a potential cell-lineage gene highly expressed in the pathogenesis of squamous cell carcinomas of the lung. PLoS One..

[14] Velcheti V, Schalper K, Yao X, Cheng H, Kocoglu M, Dhodapkar K (2013). High SOX2 levels predict better outcome in non-small cell lung carcinomas. PLoS One.

[15] Wilbertz T, Wagner P, Petersen K, Stiedl AC, Scheble VJ, Maier S (2011). SOX2 gene amplification and protein overexpression are associated with better outcome in squamous cell lung cancer. Mod Pathol..

[16] Sholl LM, Long KB, Hornick JL (2010). Sox2 expression in pulmonary non-small cell and neuroendocrine carcinomas. Appl Immunohistochem Mol Morphol..

[17] Li M, He Y, Dubois W, Wu X, Shi J, Huang J (2012). Distinct regulatory mechanisms and functions for p53-activated and p53-repressed DNA damage response genes in embryonic stem cells. Mol Cell..

[18] Chen K, Wu K, Cai S, Zhang W, Zhou J, Wang J, et al. Dachshund Binds p53 to Block the Growth of Lung Adenocarcinoma Cells. Cancer Res. 2013. 10.1158/0008-5472.CAN-12-3191 PMC3674204 23492369

[19] Jain AK, Allton K, Iacovino M, Mahen E, Milczarek RJ, Zwaka TP (2012). p53 regulates cell cycle and microRNAs to promote differentiation of human embryonic stem cells. PLoS Biol..

[20] Avgeris M, Stravodimos K, Fragoulis EG, Scorilas A (2013). The loss of the tumour-suppressor miR-145 results in the shorter disease-free survival of prostate cancer patients. Br J Cancer..

[21] Sachdeva M, Zhu S, Wu F, Wu H, Walia V, Kumar S (2009). p53 represses c-Myc through induction of the tumor suppressor miR-145. Proc Natl Acad Sci U S A..

[22] Campayo M, Navarro A, Vinolas N, Diaz T, Tejero R, Gimferrer JM (2013). Low miR-145 and high miR-367 are associated with unfavourable prognosis in resected nonsmall cell lung cancer. Eur Respir J..

[23] D'Haene B, Vandesompele J, Hellemans J (2010). Accurate and objective copy number profiling using real-time quantitative PCR. Methods..

[24] Weaver S, Dube S, Mir A, Qin J, Sun G, Ramakrishnan R (2010). Taking qPCR to a higher level: Analysis of CNV reveals the power of high throughput qPCR to enhance quantitative resolution. Methods..

[25] Lind H, Ekstrom PO, Ryberg D, Skaug V, Andreassen T, Stangeland L (2007). Frequency of TP53 mutations in relation to Arg72Pro genotypes in non small cell lung cancer. Cancer Epidemiol Biomarkers Prev..

[26] Kristensen AT, Bjorheim J, Ekstrom PO (2002). Detection of mutations in exon 8 of TP53 by temperature gradient 96-capillary array electrophoresis. Biotechniques..

[27] Zienolddiny S, Skaug V, Landvik NE, Ryberg D, Phillips DH, Houlston R (2009). The TERT-CLPTM1L lung cancer susceptibility variant associates with higher DNA adduct formation in the lung. Carcinogenesis.

[28] Ge N, Lin HX, Xiao XS, Guo L, Xu HM, Wang X (2010). Prognostic significance of Oct4 and Sox2 expression in hypopharyngeal squamous cell carcinoma. J Transl Med..

[29] Huang P, Qiu J, Li B, Hong J, Lu C, Wang L (2011). Role of Sox2 and Oct4 in predicting survival of hepatocellular carcinoma patients after hepatectomy. Clin Biochem..

[30] Matsuoka J, Yashiro M, Sakurai K, Kubo N, Tanaka H, Muguruma K (2012). Role of the stemness factors sox2, oct3/4, and nanog in gastric carcinoma. J Surg Res..

[31] Saigusa S, Mohri Y, Ohi M, Toiyama Y, Ishino Y, Okugawa Y (2011). Podoplanin and SOX2 expression in esophageal squamous cell carcinoma after neoadjuvant chemo-radiotherapy. Oncol Rep..

[32] Wang X, Liang Y, Chen Q, Xu HM, Ge N, Luo RZ (2012). Prognostic significance of SOX2 expression in nasopharyngeal carcinoma. Cancer Invest..

[33] Bass AJ, Watanabe H, Mermel CH, Yu S, Perner S, Verhaak RG (2009). SOX2 is an amplified lineage-survival oncogene in lung and esophageal squamous cell carcinomas. Nat Genet..

[34] Cai YR, Zhang HQ, Zhang ZD, Mu J, Li ZH (2011). Detection of MET and SOX2 amplification by quantitative real-time PCR in non-small cell lung carcinoma. Oncol Lett..

[35] Hussenet T, Dali S, Exinger J, Monga B, Jost B, Dembele D (2010). SOX2 is an oncogene activated by recurrent 3q26.3 amplifications in human lung squamous cell carcinomas. PLoS One.

[36] Sasaki H, Yokota K, Hikosaka Y, Moriyama S, Yano M, Fujii Y (2012). Increased Sox2 copy number in lung squamous cell carcinomas. Exp Ther Med..

[37] Nakatsugawa M, Takahashi A, Hirohashi Y, Torigoe T, Inoda S, Murase M (2011). SOX2 is overexpressed in stem-like cells of human lung adenocarcinoma and augments the tumorigenicity. Lab Invest..

[38] Chen S, Xu Y, Chen Y, Li X, Mou W, Wang L (2012). SOX2 gene regulates the transcriptional network of oncogenes and affects tumorigenesis of human lung cancer cells. PLoS One..

[39] Xiang R, Liao D, Cheng T, Zhou H, Shi Q, Chuang TS (2011). Downregulation of transcription factor SOX2 in cancer stem cells suppresses growth and metastasis of lung cancer. Br J Cancer..

[40] Singh S, Trevino J, Bora-Singhal N, Coppola D, Haura E, Altiok S (2012). EGFR/Src/Akt signaling modulates Sox2 expression and self-renewal of stem-like side-population cells in non-small cell lung cancer. Mol Cancer..

[41] McCaughan F, Pole JC, Bankier AT, Konfortov BA, Carroll B, Falzon M (2010). Progressive 3q amplification consistently targets SOX2 in preinvasive squamous lung cancer. Am J Respir Crit Care Med..

[42] Lu Y, Futtner C, Rock JR, Xu X, Whitworth W, Hogan BL (2010). Evidence that SOX2 overexpression is oncogenic in the lung. PLoS One..

[43] Brcic L, Sherer CK, Shuai Y, Hornick JL, Chirieac LR, Dacic S (2012). Morphologic and Clinicopathologic Features of Lung Squamous Cell Carcinomas Expressing Sox2. Am J Clin Pathol..

[44] Sholl LM, Barletta JA, Yeap BY, Chirieac LR, Hornick JL (2010). Sox2 protein expression is an independent poor prognostic indicator in stage I lung adenocarcinoma. Am J Surg Pathol..

[45] Hussenet T, du Manoir S (2010). SOX2 in squamous cell carcinoma: amplifying a pleiotropic oncogene along carcinogenesis. Cell Cycle..

[46] Toschi L, Finocchiaro G, Nguyen TT, Skokan MC, Giordano L, Gianoncelli L (2014). Increased SOX2 Gene Copy Number Is Associated with FGFR1 and PIK3CA Gene Gain in Non-Small Cell Lung Cancer and Predicts Improved Survival in Early Stage Disease. PLoS One..

[47] Chen Y, Huang Y, Huang Y, Chen J, Wang S, Zhou J (2013). The prognostic value of SOX2 expression in non-small cell lung cancer: a meta-analysis. PLoS One.

[48] Shlien A, Tabori U, Marshall CR, Pienkowska M, Feuk L, Novokmet A (2008). Excessive genomic DNA copy number variation in the Li-Fraumeni cancer predisposition syndrome. Proc Natl Acad Sci U S A..

[49] Husgafvel-Pursiainen K, Boffetta P, Kannio A, Nyberg F, Pershagen G, Mukeria A (2000). p53 mutations and exposure to environmental tobacco smoke in a multicenter study on lung cancer. Cancer Res..

[50] Takagi Y, Osada H, Kuroishi T, Mitsudomi T, Kondo M, Niimi T (1998). p53 mutations in non-small-cell lung cancers occurring in individuals without a past history of active smoking. Br J Cancer..

[51] Vahakangas KH, Bennett WP, Castren K, Welsh JA, Khan MA, Blomeke B (2001). p53 and K-ras mutations in lung cancers from former and never-smoking women. Cancer Res..

[52] MacNeil AJ, Jiao S-C, McEachern LA, Yang YJ, Dennis A, Yu H (2014). MAPK Kinase 3 Is a Tumor Suppressor with Reduced Copy Number in Breast Cancer. Cancer Res..

[53] Fischer U, Heckel D, Michel A, Janka M, Hulsebos T, Meese E (1997). Cloning of a Novel Transcription Factor-Like Gene Amplified in Human Glioma Including Astrocytoma Grade I. Hum Mol Genet..

[54] Valinezhad Orang A, Safaralizadeh R, Kazemzadeh-Bavili M (2014). Mechanisms of miRNA-Mediated Gene Regulation from Common Downregulation to mRNA-Specific Upregulation. Int J Genomics..

[55] Cordes KR, Sheehy NT, White M, Berry E, Morton SU, Muth AN (2009). miR-145 and miR-143 Regulate Smooth Muscle Cell Fate Decisions. Nature.

[56] Li E, Zhang J, Yuan T, Ma B (2014). miR-145 inhibits osteosarcoma cells proliferation and invasion by targeting ROCK1. Tumor Biology.

[57] Park JH, Roeder RG (2006). GAS41 is required for repression of the p53 tumor suppressor pathway during normal cellular proliferation. Mol Cell Biol..

[58] Han J, Sun P (2007). The pathways to tumor suppression via route p38. Trends Biochem Sci..

