# Supplementary material for: Mutations in TP53 increase the risk of SOX2 copy number alterations and silencing of TP53 reduces SOX2 expression in non-small cell lung cancer
Source: BMC Cancer. 2016 Jan 19;16:28. doi: 10.1186/s12885-016-2061-3 (PMC4717590; doi:10.1186/s12885-016-2061-3)
Supplement: Additional file 1: Table S1. — Association between clinicopathological data and SOX2 gene status in NSCLC tumors. (PDF 101 kb) [file 12885_2016_2061_MOESM1_ESM.pdf]

Suppl. table 1. Association between clinicopathological data and gene status in tumor tissues.

	SOX2 gene status		
	normal	***CNV****	***p-value***[†]
Age			
<55	26	16	ns
≥55	122	82	
Gender			
Female	47	24	ns
Male	101	74	
Smoking			
Non-smokers	15	2	p=0.015
Smokers	126	90	
Pack-years[‡]			
≤20	40	31	ns
>20,≤40	52	33	
>40	36	24	

*Samples were defined to have a copy number variation (CNV) if the copy number was above 2.5 or below 1.5. [†]p-values for categorical variables were obtained from Chi-square or Fisher's exact test and for ordinal variables from nonparametric Mann-Whitney test or Kruskal Wallis ANOVA. [‡]Pack-years indicates the number of cigarettes smoked per day x number of years smoked/20.
